# Antioxidant Effect of *Polygonatum sibiricum* Polysaccharides in D-Galactose-Induced Heart Aging Mice

**DOI:** 10.1155/2021/6688855

**Published:** 2021-03-29

**Authors:** Wanjun Ma, Shanshan Wei, Weijun Peng, Taoli Sun, Jianhua Huang, Rong Yu, Bikui Zhang, Wenqun Li

**Affiliations:** ^1^Department of Pharmacy, The Second Xiangya Hospital, Central South University, Changsha, Hunan 410011, China; ^2^Institute of Clinical Pharmacy, Central South University, Changsha, Hunan 410011, China; ^3^Department of Integrated Traditional Chinese & Western Medicine, The Second Xiangya Hospital, Central South University, Changsha, Hunan 410011, China; ^4^Key Laboratory of Hu'nan Oriented Fundamental and Applied Research of Innovative Pharmaceutics, College of Pharmacy, Changsha Medical University, Changsha, Hunan 410219, China; ^5^Hunan Academy of Chinese Medicine, Hunan University of Chinese Medicine, Changsha, Hunan 410013, China; ^6^Hunan Key Laboratory of TCM Prescription and Syndromes Translational Medicine, Changsha, Hunan 410208, China

## Abstract

*Polygonatum sibiricum* polysaccharides (PSP), the extract of *Polygonatum sibiricum*, are demonstrated to exhibit a wide range of pharmacological activities. A recent study reported that PSP alleviated the aging of the kidney and meninges. However, the effect of PSP on heart aging remains unclear. The present study is aimed at investigating the protection of PSP on D-galactose- (D-gal-) induced heart aging. Results showed that irregularly arranged cardiac muscle fibers were observed in heart tissues of D-gal-treated mice, and the levels of cardiac troponin T (cTnT), creatine kinase (CK), p21, and p53 were increased after D-gal treatment. D-gal-induced heart aging and injury can be attenuated by oral administration of PSP. Moreover, PSP also decreased reactive oxygen species (ROS) and malondialdehyde (MDA) and increased the level of superoxide dismutase (SOD) in the hearts of D-gal-treated mice. DNA damages and lipid peroxidation induced by oxidative stress were also inhibited by PSP as indicated by reduced levels of 8-hydroxydeoxyguanosine (8-OHdG) and 4-hydroxy-2-nonenal (4-HNE). Collectively, PSP attenuated D-gal-induced heart aging via inhibiting oxidative stress, suggesting that PSP might serve as a potential effective Chinese herbal active constituent for antiaging therapy.

## 1. Introduction

As a common and inevitable natural phenomenon, aging is an ongoing physiological process that is accompanied by material, morphological, and functional degradation in cells, tissues, and organs [1]. However, symptoms of aging do not show up until one is at the specific age. Public concern has been raised regarding the potential health risks of aging and the protection of body degeneration since population aging has become a global trend. Clinical statistics exhibited that morbidities and mortalities of heart diseases are apparently higher in the elderly people when compared with the youths [2]. Over the past few decades, researchers have concluded that heart aging tended to give rise to cardiovascular diseases such as myocardial ischemia, atherosclerosis, and cardiac fibrosis, which finally ended up with heart failure. Moreover, aging has also been acknowledged as a risk factor of cardiovascular surgery [3]. Therefore, there is an urgent need to identify novel antiaging agents to alleviate aging-related heart injuries and improve the health of elderly people.

Emerging evidence indicates that natural active substance or components isolated from plants could be candidate therapeutic agents for the prevention and treatment of aging-related diseases. Flavonoids, terpenoids, saponins, and polysaccharides are currently the most representative phytochemicals in preventing decrepitude [4]. *In vitro* experiment demonstrated that *Ginkgo biloba* extract distinctly delayed the aging of endothelial progenitor cells by activating telomerase, a critical target in antiaging treatment [5]. Additionally, *in vivo* experiments further elucidated the protective effects of natural active components, such as resveratrol, hyperoside, and 4,4′-dimethoxychalcone [6–8]. More importantly, clinical evaluation for antiaging activity of resveratrol has been performed in an experiment made up of 97 healthy human subjects (62 males and 35 females), and the antiaging activity of resveratrol was proved to be related to the inhibition on age-dependent oxidative stress [9].

*Polygonatum sibiricum* is a biologically active Chinese herb that has wide usage among food and medical industries. *Polygonatum sibiricum* polysaccharide (PSP), the major pharmacology active constituent isolated from *Polygonatum sibiricum*, is widely applied to the therapy of cardiovascular diseases or osteoporosis [10, 11]. Recently, PSP was reported to have an antiaging effect in the kidney and meninges, and it can alleviate kidney injury and declines of learning ability induced by D-galactose (D-gal) [12]. However, the effect of PSP on heart aging has not been investigated to date. Therefore, the present study was designed to investigate and evaluate the antiaging activity of PSP on D-gal-induced heart aging and injury *in vivo* and determine whether the underlying mechanism involved oxidative stress.

## 2. Materials and Methods

### 2.1. Drugs and Reagents

*Polygonatum sibiricum* polysaccharides (PSP) were purchased from YuanYe Bio-Technology Co., Ltd. (Shanghai, China) with a purity of 70%. D-gal (purity > 98%) was purchased from Nanjiang Pharmaceutical Co., Ltd. (Kunming, China). Aging *β*-galactosidase staining kit, total superoxide dismutase (SOD) assay kit, malondialdehyde (MDA) assay kit, and reactive oxygen species (ROS) assay kit were purchased from Beyotime Biotechnology Co., Ltd. (Shanghai, China). The antibodies were purchased from Abcam (Cambridge, USA); EasySee Western Blotting Kit was purchased from Beijing TransGen Biotech Co., Ltd. (Beijing, China).

### 2.2. Animals and Treatments

Kunming mice (male, 6-8 weeks old, weighing 20 ± 2 g) were obtained from the Laboratory Animal Center, Xiangya School of Medicine, Central South University (Changsha, China). All experiments were conducted according to the National Institutes of Health Guide (NIH publications no. 8023) for the Care and Use of Laboratory Animals. The experimental protocol was approved by the Medicine Animal Welfare Committee of Xiangya School of Medicine (No. SYXK-2016/0002).

The mice were randomly divided into 4 groups (6 mice in each group) and treated as follows: (i) control group (saline, i.g.), (ii) D-gal group (500 mg/kg/d for 60 days, i.h.), (iii) low-dose PSP (200 mg/kg/d for 60 days, i.g.) plus D-gal (500 mg/kg/d for 60 days, i.h.), and (iv) high-dose PSP (400 mg/kg/d for 60 days, i.g.) plus D-gal (500 mg/kg/d for 60 days, i.h.). The dosages of PSP and D-gal were determined according to previous studies [13, 14]. All animals were anaesthetized by intraperitoneal injection of 1% pentobarbital sodium (50 mg/kg, i.p.). Blood samples were obtained by orbital blood collection. Hearts without atria were dissected and rinsed with cold saline. A portion of the heart was preserved in 10% formalin for histology. The remaining portion of the heart was quickly frozen in liquid nitrogen and then stored at −80°C for further biochemical analysis.

### 2.3. Immunohistochemistry

Immunohistochemistry of the heart was conducted according to our previous study [15]. The hearts were embedded in the paraffin and cut into sections (5 *μ*m). The endogenous peroxidase activity was quenched by treating with 0.3% H_2_O_2_ and then incubated with 1% Triton X-100% and 1% normal rabbit serum for 2 h. The slices were infiltrated with the appropriate primary antibodies (p21, 4-HNE, and 8-OHdG) at 4°C overnight and then incubated with the second antibody for about 2 h. The positive signal was detected through color reaction caused by diaminobenzidine. A digital camera was used to capture immunohistochemical images.

### 2.4. Creatine Kinase (CK) and Cardiac Troponin T (cTnT) Measurement

The coagulated blood samples collected from animals were centrifuged at 4000 rpm in 4°C for 15 min to obtain serum. The serum biochemical parameters including cTnT and CK were analyzed by using kits with an automatic biochemical analyzer (Abbott Pharmaceutical Co., Ltd., Lake Bluff, IL, USA). The determination of CK and cTnT was conducted in accordance with the manufacturer's instructions.

### 2.5. *β*-Galactosidase Staining

The *β*-galactosidase staining was conducted by using a commercial kit according to the manufacturer's instructions and our previous study [15]. Heart slices were infiltrated by *β*-galactosidase staining working fluid at 37°C overnight and then washed by PBS. 10 random images from each sample were captured by a digital camera.

### 2.6. Western Blotting

Heart tissue was grinded to extract the protein with RIPA buffer (containing 0.1% PMSF). Equivalent amount of protein was separated and transferred into polyvinylidene fluoride membrane by 10% or 12% sodium dodecyl sulfate polyacrylamide gel electrophoresis. The membranes were blocked with 5% nonfat milk for 1 h, incubated with the primary antibodies overnight at 4°C, and then probed with the corresponding secondary antibody for 1 h at room temperature. The chemiluminescence signals and band intensities were analyzed by ImageJ 1.43 (National Institutes of Health) with the EasySee Western Blotting Kit. The dilution ratios of the primary antibodies were shown as follows: p21 (ab109199, 1 : 1000, Abcam), p53 (ab131442, 1 : 1000, Abcam), 4-HNE (ab46545, 1 : 1000, Abcam), and GAPDH (sc-137179, 1 : 2000, Santa Cruz).

### 2.7. ROS Measurement

The fluorescent probe dihydroethidium (DHE) was used to monitor intracellular ROS levels. Intracellular DHE is oxidized to ethidium, which binds to DNA and stains the nuclei bright fluorescent red. The detection of ROS level in the heart section was conducted according to the manufacturer's instructions.

### 2.8. MDA Content and SOD Activity Measurement

The levels of MDA and SOD activity were quantified according to the manufacturer's protocol. In brief, heart tissue extracts were prepared by sonication in ice-cold buffer (50 mM Tris-HCl, pH 7.5, 5 mM EDTA, and 1 mM DTT). After sonication, lysed cells were centrifuged at 10000 × g for 20 min to remove debris. The supernatant was subjected to the measurement of MDA and SOD levels and the protein contents. The absorbance (OD) was determined by a microplate reader at 532 nm (MDA) and 450 nm (SOD). MDA and SOD levels were normalized to milligram protein.

### 2.9. Statistical Analysis

All data are expressed as mean ± SEM. Unpaired Student's *t*-test for two comparisons or ANOVA followed by the Student–Newman–Keuls test for multiple comparisons was conducted to perform statistical analysis. *P* < 0.05 was considered statistically significant.

## 3. Results

### 3.1. PSP Attenuates the Myocardial Injury Induced by D-gal in Mice

**Figure 1:**
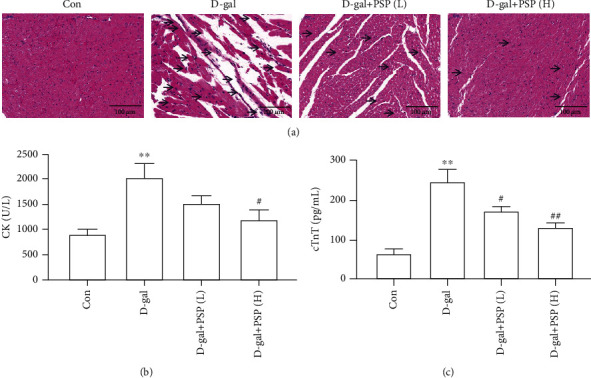
Effect of PSP on D-galactose-induced myocardial injury in mice. (a) Representative images of HE staining in the myocardium (magnification, ×200), inflammatory infiltrations and disorder of cardiac fiber arrangement were marked by arrows. (b, c) The level of myocardial enzymes CK and cTnT in plasma. PSP (L): 200 mg/kg/d, PSP (H): 400 mg/kg/d. D-gal: D-galactose; PSP: *Polygonatum sibiricum* polysaccharides; CK: creatine kinase; cTnT: cardiac troponin T. Data are mean ± SEM; *n* = 6. ^∗∗^*P* < 0.01 vs. Con; ^#^*P* < 0.05 and ^##^*P* < 0.01 vs. D-gal.

D-gal-induced myocardial injury is a canonical model used to investigate heart aging. HE staining was conducted to detect the myocardial morphology alterations. Results showed inflammatory infiltrations and disorder of cardiac fiber arrangement in D-gal-treated mice, which can be attenuated by PSP administration (Figure 1(a)). Moreover, myocardial enzymes such as CK and cTnT were measured to investigate myocardial injuries. CK and cTnT levels in plasma were tested as shown in Figures 1(b) and 1(c). We found that the levels of CK and cTnT in plasma were increased in D-gal-treated mice, and PSP treatment can apparently decrease those specific myocardial enzyme levels, suggesting that D-gal-induced myocardial injury was attenuated by PSP.

### 3.2. PSP Protects the Heart against D-gal-Induced Aging in Mice

**Figure 2:**
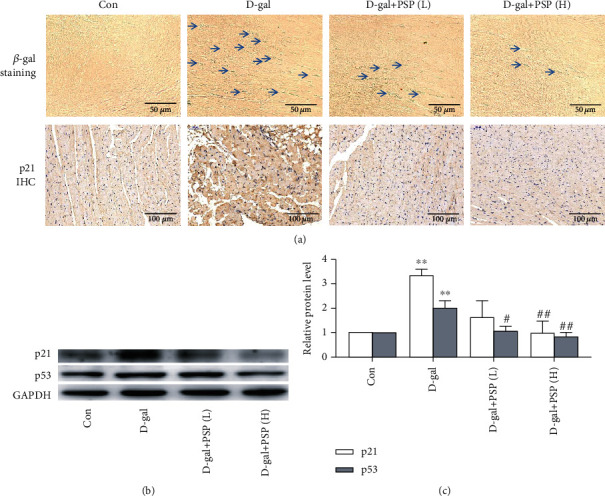
Effect of PSP on D-galactose-induced myocardial aging in mice. (a) Representative images of *β*-galactosidase staining and p21 immunohistochemistry in the myocardium, and the *β*-galactosidase staining-positive cells were indicated by arrows (magnification, ×400 for *β*-galactosidase staining and ×200 for p21 IHC). (b, c) The protein expressions of p21 and p53 in the myocardium. Data are mean ± SEM; *n* = 6. ^∗∗^*P* < 0.01 vs. Con; ^#^*P* < 0.05 and ^##^*P* < 0.01 vs. D-gal.

It has been widely accepted that aging is closely related to the upregulation of p21 and p53 expression; the presence of *β*-galactosidase-positive cells also reflects aging. Therefore, we conducted *β*-galactosidase staining and p21 immunohistochemistry in the myocardium to determine the heart aging level. Results displayed increased p21 expression and *β*-galactosidase-positive cells in the myocardium of D-gal-treated mice, which can be reversed by PSP treatment (Figure 2(a)). Moreover, upregulation of p21 and p53 protein expressions was also observed in D-gal-treated mice, and PSP reversed these alterations (Figures 2(b) and 2(c)). These results indicated the establishment of heart aging model, whereas PSP administration can inhibit the heart aging induced by D-gal (Figures 2(a)–2(c)).

### 3.3. PSP Inhibits the Myocardial Oxidative Stress Induced by D-gal in Mice

**Figure 3:**
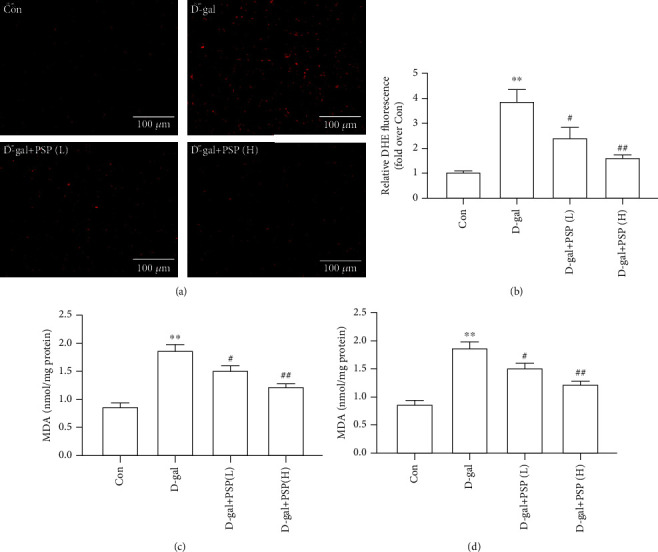
Effect of PSP on myocardial oxidative stress induced by D-galactose in mice. (a, b) Representative images of DHE fluorescence for determining ROS level (magnification, ×200). (c, d) The contents of MDA and ROS in the myocardium. Data are mean ± SEM; *n* = 6. ^∗∗^*P* < 0.01 vs. Con; ^#^*P* < 0.05 and ^##^*P* < 0.01 vs. D-gal.

Oxidative stress plays a key role in heart aging, and the overproduction of ROS is recognized as the typical character. As shown in Figures 3(a) and 3(b), the ROS fluorescent probe DHE was used to measure ROS level in the myocardium, and the result showed that the myocardial ROS level was increased in D-gal-treated mice. Antioxidant enzyme SOD is responsible for the balance of prooxidant and antioxidant during oxidative stress, and lipid oxidation product MDA also reflects the oxidative stress level. The results showed that D-gal increased the MDA content and decreased the SOD content (Figures 3(c) and 3(d)), suggesting the elevation of oxidative stress level. However, PSP administration inhibited the myocardial oxidative stress induced by D-gal as indicated by decreased ROS level and MDA content, as well as increased SOD content (Figures 3(a)–3(d)).

### 3.4. PSP Alleviates the Oxidative Stress-Induced Myocardial Lipid Peroxidation and DNA Damage in D-gal-Treated Mice

**Figure 4:**
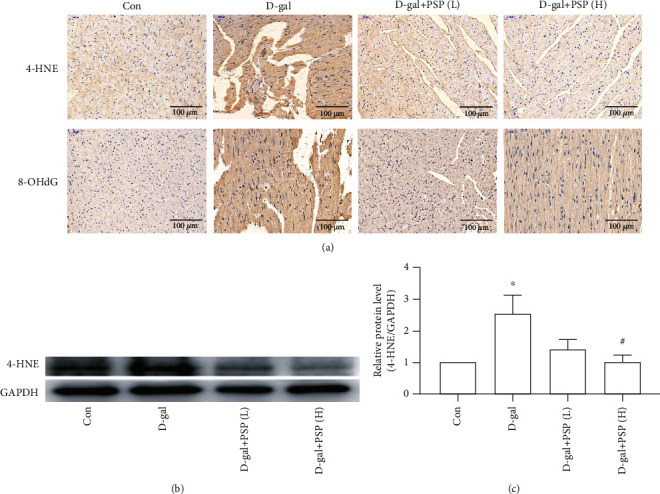
Effect of PSP on the myocardial lipid peroxidation and DNA damage in D-galactose-treated mice. (a) Representative images of 4-HNE and 8-OHdG immunohistochemistry in the myocardium (magnification, ×200). (b, c) The 4-HNE protein expression of the myocardium. Data are mean ± SEM; *n* = 6. ^∗^*P* < 0.05 vs. Con; ^#^*P* < 0.05 and ^##^*P* < 0.01 vs. D-gal.

Increased oxidative stress induces lipid peroxidation and DNA damage, which finally leads to heart aging and injury. To further confirm the protective role of PSP against D-gal-induced heart aging and injury, we investigated lipid peroxidation and DNA damage. 4-HNE and 8-OHdG are the typical markers that indicate lipid peroxidation and DNA damage, respectively. The result of immunohistochemistry indicated upregulations of myocardial 4-HNE and 8-OHdG in D-gal-treated mice, whereas PSP administration alleviated the effect of D-gal (Figure 4(a)). The consistent result was found in 4-HNE protein expression that is measured by western blotting (Figures 4(a)–4(c)). Collectively, these results suggested that PSP can alleviate the oxidative stress-induced myocardial lipid peroxidation and DNA damage in D-gal-treated mice.

## 4. Discussion

PSP is a pharmacology active constituent that is isolated from the dry rhizome of traditional oriental medicine, *Polygonatum sibiricum* [16]. With in-depth studies, researchers discovered that PSP exhibits a variety of biological activities, such as antiaging, reducing blood lipids, enhancing immune function, and promoting osteoblastic differentiation [10, 17, 18]. Recently, it has been reported that PSP administration inhibited acute heart failure and cardiomyocyte death induced by Adriamycin insult, indicating the protection of PSP on the heart [19].

In recent years, an increasing number of studies demonstrated that the morbidity and mortality of cardiovascular diseases are raised accompanying with aging, and growing public concern has been improved regarding the potential health risks of elderly people. To investigate the mechanisms of individual aging and to evaluate the curative effects of antiaging drugs at the integral organ and tissue level, several experimental aging animal models are developed into research. D-gal is the currently prevalent proaging agent [20]. Song et al. firstly reported that a low dose of D-gal injection could induce an aging-accelerating effect on mice. D-gal formed advanced glycation end products in vivo, and the elevated advanced glycation end products may accelerate the aging process [21]. Moreover, free radical disorders, mitochondrial dysfunction, and cell injury are observed in D-gal-treated mice, which are similar to natural aging ones [20]. The aging-related genes including p16 and p21 were also increased in cardiomyocytes under D-gal treatment [22]. Collectively, the promotion of the advanced glycation end products derived from D-gal on aging process may attribute its effects on oxidative stress and aging-related genes.

Therefore, we established aging model mice by subcutaneous injections of D-gal (500 mg/kg/d) for 60 days. The D-gal group exhibited a distinct reduction of movement frequency and rough fur in apparent behavior when compared with the control group, indicating the proaging effect of D-gal insult. Up to date, an accumulating number of biochemical indexes have been developed to evaluate heart injury. In particular, CK and cTnT have always been regarded as canonical and reliable in clinically estimating heart injury since the last century [23]. Irregularly arranged cardiac muscle fibers and increased levels of CK and cTnT in plasma were discovered in D-gal-treated mice. *β*-Galactosidase staining is widely recognized as an effective measurement and evidence for aging [24]. Results showed that there were more *β*-galactosidase-positive cells in the D-gal group than the control group. Additionally, cell cycle arrest is the important indicator of irreversible aging. Telomere dysfunction and DNA damage were demonstrated the initiation of cell cycle arrest [25]. As one of the downstream effectors of DNA damage, tumor-inhibiting factor p53 upregulates cell cycle regulatory protein p21 to arrest cells in the G1 phase of the cell cycle [26, 27]. In the present study, immunohistochemical results displayed that the expression of p53 and p21 was upregulated in the D-gal group, suggesting that D-gal did induce heart aging in mice.

To the best of our knowledge, PSP could significantly improve the learning and memory abilities and reverse the pathological changes of kidney tissues in D-gal-induced natural aging-like model mice, suggesting the antiaging effect of PSP on D-gal-induced kidney and meninges aging [12]. The present study further demonstrated that the D-gal-induced heart aging and injury can be attenuated by oral administration of PSP. It has been reported that PSP attenuates the aging of the kidney and meninges by regulating the Klotho-FGF23 endocrine axis, alleviating oxidative stress, and balancing calcium and phosphorus metabolism. However, the mechanism underlying the inhibition of PSP on heart aging remains unclear.

Accumulating evidences support the hypothesis that the consistent oxidative stress injury is one of the main causes of aging [28, 29]. ROS-induced inflammation, mitochondrial dysfunction, and ion disorder generate oxidative damage and aging-related phenotypes in hearts [30, 31]. Moreover, the antioxidant enzymes in injured hearts were decreased, which somehow further exacerbates oxidative damage [32]. As an important factor of aging, ROS accumulation contributes to the occurrence and development of a series of cardiovascular diseases [33]. MDA and SOD are critical indexes to reflect oxidative stress activity in cells. As the production of lipid peroxidation had arisen by free radical action, MDA contributes to the disorder of biomacromolecule, involving nucleic acid, protein, and phospholipid in the process of aging [34]. SOD is the essential oxygen free radical removal enzyme *in vivo*, which further turns superoxide anion radical O_2_^−^ into H_2_O_2_ and O_2_ to prevent against oxidative stress injuries [35]. In the present study, the levels of ROS and MAD were increased and the SOD level was decreased in the heart after D-gal treatment, indicating D-gal enhanced oxidative stress in the heart to accelerate aging. Since the antioxidative activity of PSP has been uncovered, it would be worthwhile and interesting to investigate the regulation of PSP on oxidative stress in D-gal-induced natural aging-like model mice. We found that PSP significantly decreased the levels of ROS and MDA and increased the level of SOD. This finding indicated that PSP attenuated heart aging via inhibiting oxidative stress injury.

It is well known that ROS were mainly produced by electron transport in the respiration reaction occurring in mitochondria. DNA damage caused by ROS accumulation is considered as an important cause of aging. ROS could target the nucleotide pool to elevate the expressions of p21, p53, and p16 and eventually accelerate the process of cell aging [36]. Moreover, excessive oxidative stress breaks the redox balance of the mitochondria, induces lipid peroxidation, and damages the membrane structures of the mitochondria, endoplasmic reticulum, and cell itself, gradually resulting in aging and functional regression [28]. The present study found that PSP could suppress the lipid peroxidation and DNA damage of the heart in D-gal-induced aging mice, which further confirmed that PSP attenuates heart aging via inhibiting oxidative stress.

However, several limitations have to be recognized in this study. The lack of PSP alone group may raise the doubt for PSP safety, despite the polysaccharide being of benefit to the body in general. Moreover, the in vitro experiment is not conducted to validate the conclusion in vivo, and the detailed mechanism in the antiaging effect of PSP also deserved exploration in vitro, which will be the focus in our further study.

## 5. Conclusion

In conclusion, the present study firstly demonstrates the cardioprotective effect of PSP on the D-gal-induced natural aging-like model mice. The underlying mechanism appears to involve the inhibition of oxidative stress in the heart. These results may provide a scientific basis supporting further use of PSP in antiaging treatment.

## Data Availability

The data used to support the findings of this study are available from the corresponding author upon request.
