# Corrigendum to “Antioxidant Effect of *Polygonatum sibiricum* Polysaccharides in D-Galactose-Induced Heart Aging Mice”

**DOI:** 10.1155/2021/9806412

**Published:** 2021-06-03

**Authors:** Wanjun Ma, Shanshan Wei, Weijun Peng, Taoli Sun, Jianhua Huang, Rong Yu, Bikui Zhang, Wenqun Li

**Affiliations:** ^1^Department of Pharmacy, The Second Xiangya Hospital, Central South University, Changsha, Hunan 410011, China; ^2^Institute of Clinical Pharmacy, Central South University, Changsha, Hunan 410011, China; ^3^Department of Integrated Traditional Chinese & Western Medicine, The Second Xiangya Hospital, Central South University, Changsha, Hunan 410011, China; ^4^Key Laboratory of Hu'nan Oriented Fundamental and Applied Research of Innovative Pharmaceutics, College of Pharmacy, Changsha Medical University, Changsha, Hunan 410219, China; ^5^Hunan Academy of Chinese Medicine, Hunan University of Chinese Medicine, Changsha, Hunan 410013, China; ^6^Hunan Key Laboratory of TCM Prescription and Syndromes Translational Medicine, Changsha, Hunan 410208, China

In the article titled “Antioxidant Effect of *Polygonatum sibiricum* Polysaccharides in D-Galactose-Induced Heart Aging Mice” [1], there is an error in Figure 3 where Figure 3(d) is incorrectly duplicated with Figure 3(c) due to an error in the production process. Additionally, in the legend of Figure 3, “The contents of MDA and ROS in the myocardium” should be corrected to “The contents of MDA and SOD in the myocardium”. The authors confirm that this does not affect the results or conclusions of the article, and the corrected figure and legend are as follows.

## Figures and Tables

**Figure 1:**
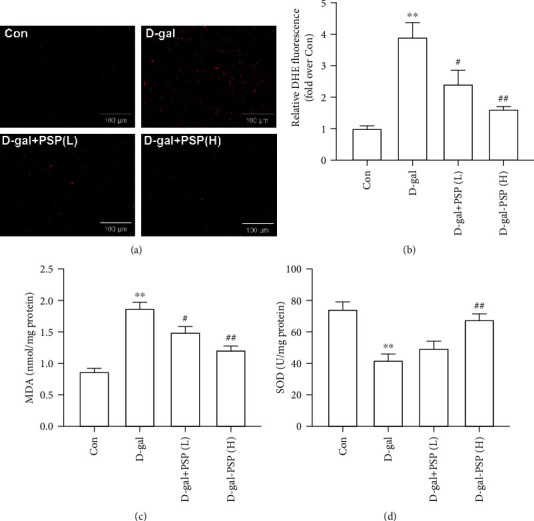
Effect of PSP on myocardial oxidative stress induced by D-galactose in mice. (a, b) Representative images of DHE fluorescence for determining ROS level (magnification, ×200); (c, d) the contents of MDA and SOD in the myocardium. Date are mean ± S.E.M.*n* = 6. ^∗∗^*P* < 0.01 vs. Con; ^#^*P* < 0.05 and ^##^*P* < 0.01 vs. D-gal.
